# In Tribute to Stefan Kunz

**DOI:** 10.3390/v13091840

**Published:** 2021-09-15

**Authors:** Mar Perez

**Affiliations:** Spanish Ministry of Defense, Paseo de la Castellana, 109, 28071 Madrid, Spain; mapema@gmail.com

We do not always remember the exact moment in which we first met our friends. However, I perfectly recall the day I talked to Stefan for the very first time. Or, to be precise, the first time that Stefan talked to me as I was desperately trying not to choke on a glass of water. It was Stefan’s first day at TSRI and he had set out on a mission to introduce himself to every other person in the Viral-Immunology lab, where I had started my postdoc a couple of months before. There was no way that I could have known at the time, but I had just met a superb colleague, a remarkable scientist, a generous teacher, a charismatic mentor, and a great friend. But there he was, just standing right in front of a water cooler.

The first thing everyone noticed about Stefan is that he dressed in black almost every day or, if feeling particularly daring, in jeans and a white t-shirt. When asked about his limited choice of colours, he would hide behind the claim of being partially colour-blind. Nevertheless, it was quite evident that, for him, life was too short, and brimful of interesting projects as to waste even a second of time on petty decisions.

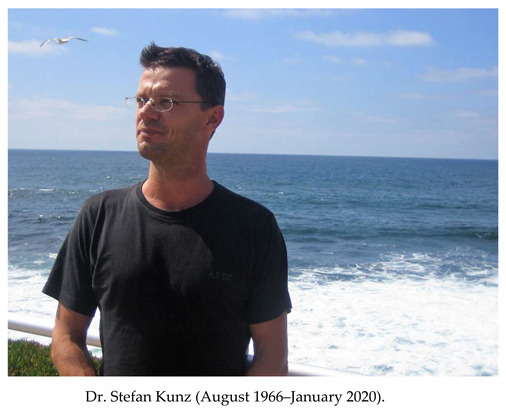



As a true Swiss, Stefan deeply enjoyed being outdoors, hiking, running, or skiing, but he also loved travelling, reading, and of course, cooking. He used to say that his Italian genes made him extremely fond of good food but had not taught him how to cook, so he had to fix that by taking cooking classes. Those fortunate enough to try his cooking would agree that the lessons really paid off. If you do not believe me, ask Nathalie, Kurt, Noemí, Elina, Hanna, or any of the many friends that he made while working in San Diego. At home, my husband and I still regularly prepare the recipes he shared with us.

Stefan had been gifted with a bright mind and boundless energy that he put to work for the benefit of science and society. Despite his love for research, he was a man of many interests, who could talk about the Bauhaus or a virus entry mechanism with the exact same ease and passion. He was always eager to learn, be it a new immunological technique, another language (he inadvertently insulted me a few times while practicing his Spanish), or even a step of salsa (although truth be told, he was a terrible dancer).

Over the years, we had endless chats about every single topic, heated scientific discussions at virology meetings, and many trips and meals. When the time came to marry the love of his life, Karin, he asked me to be his best man, notwithstanding the fact that I am a woman, which, in my opinion, is a great example of Stefan’s personality. Conventionalism never got in his way!

In passing away, Stefan leaves an enormous void, both in the scientific community and in the lives of his friends and family. His contributions to the field of virology are well known, but what his friends and colleagues will remember is the integrity and human warmth of his personality.

At his funeral, his friend Leonard read one of Stefan’s favourite poems, “The Palace” by Rudyard Kipling. This beautiful text perfectly reflects Stefan’s vision on science, a worldwide collaborative effort in which every achievement, big or small, rests on the work of other scientists with whom we share the passion for discovery. Therefore, it just feels right to also share here its last line: “After me cometh a Builder. Tell him, I too have known!”

“Hasta siempre, suizo loco”.

(So long, crazy Swiss).

